# Aspects of a circular bioeconomy: a note on milk and egg byproducts

**DOI:** 10.1093/af/vfaf023

**Published:** 2025-09-19

**Authors:** Stewart Ledgard, Michael R F Lee, David Meo Zilio, Philippe Becquet

**Affiliations:** AgResearch Limited, Ruakura Research Centre, Hamilton, New Zealand; School of Sustainable Food and Farming, Harper Adams University, Newport, UK; CREA, Research Centre for Animal Production and Aquaculture, Rome, Italy; Philippe Becquet EI, Mulhouse, France

**Implications**

- Milk and eggs are primarily used for human consumption, either directly (e.g., raw milk and table eggs) or following processing (e.g., pasteurized milk and hydrated egg powder).
- Milk is also further processed into high-value dairy products (e.g., butter, cheese, and yoghurt) primarily for direct human consumption. Whey is the major by-product of milk processing, and high-value foods can be obtained from it (e.g., whey protein concentrates for sports nutrition). However, more upcycling options for human consumption are needed globally to maintain its nutrients within the food chain and improve sustainability, via circularity.
- Eggs are also further processed into specific products for the food industry (e.g., liquid eggs, albumen, and yolk). Eggshell and membranes are the major by-products from this process, containing a high level of minerals (eggshell) and high-value protein (egg membrane). As such, eggshells and membranes may be used to maintain these nutrients within the food chain and improve sustainability via circularity.

## Introduction

**Figure 1.**
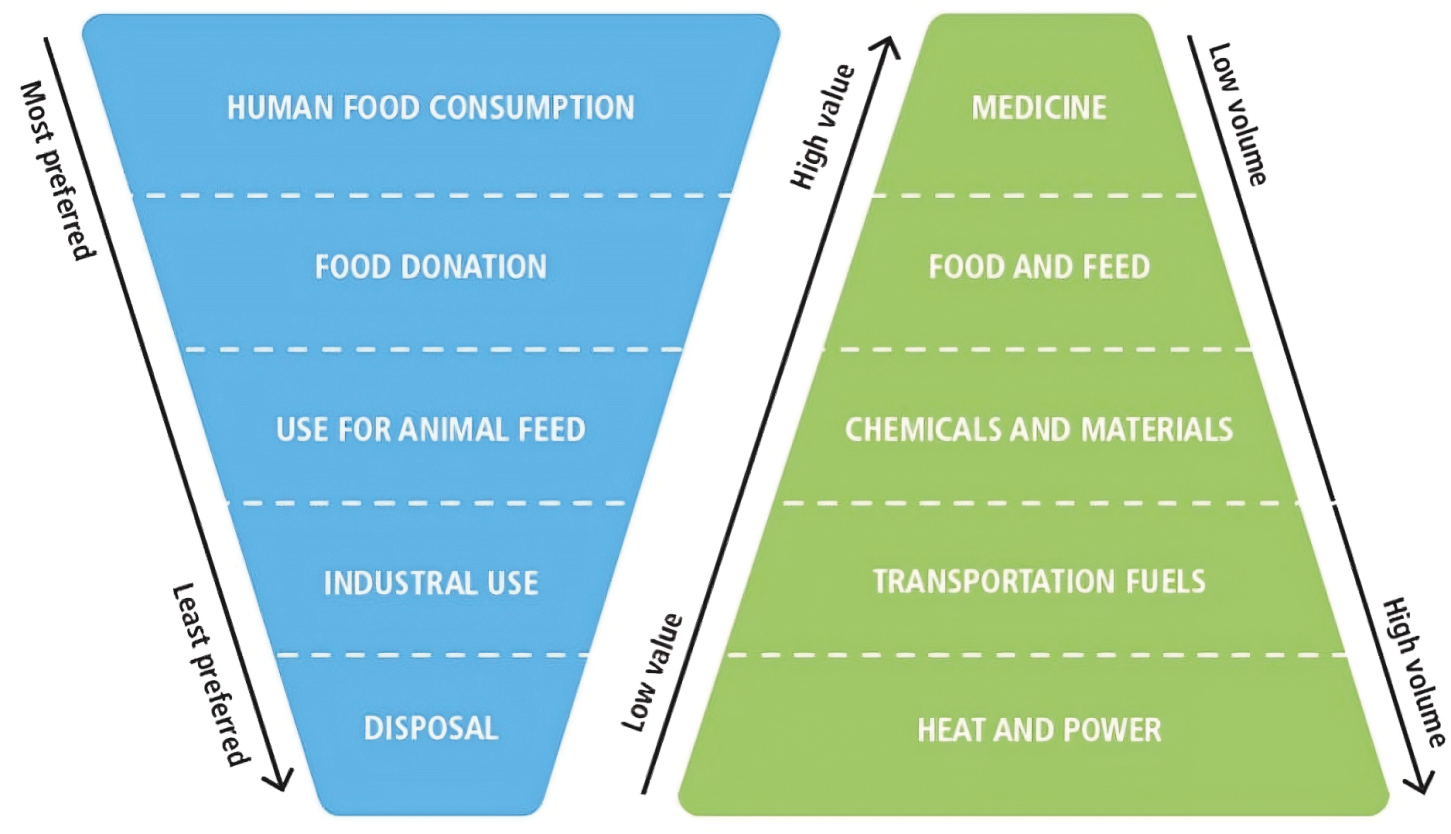
Milk and egg co-products hierarchy and value pyramid. Source: (Al-Zohairi et al., 2023).

Livestock production is developed globally with the aim of producing animal-sourced food such as milk and eggs, which are consumed directly (e.g., liquid milk and table eggs). Due to its natural microbial and oxidative characteristics, milk needs to be either consumed within a short period post-collection, following pasteurization, from the farm gate, or preserved for consumption in developed food chains. Pasteurization via heat treatment is a requirement to ensure microbial safety of the product (e.g., *Mycobacterium bovis*), although the process also extends the shelf-life of the product, as does microfiltration, which is increasingly used in longer shelf-life brands. More extreme heat treatments, such as ultra-high temperature processes, are designed specifically to address shelf-life issues of liquid milk. The formation of yoghurt via fermentation will also preserve the nutritive quality of milk and extend the shelf-life, producing a distinctive product. Furthermore, milk and eggs are further processed for producing high-value products, such as butter and a whole array of cheeses, or liquid eggs, further separated into albumen and egg yolk. Further processing of milk and eggs leads to the production of by-products, which may be used within various industries. In a circular bioeconomy, the maintenance of nutrients in the food value chain is a key objective and the concept of valorization of these byproducts follows the principle of “Food Waste Hierarchy” and “Value Pyramid” described by Al Zohairi et al. (2023) (Figure 1). Sometimes, these concepts are described as a “cascading use of biomass” (Dubois and Gomez San Juan, 2016). The preferred use of by-products from dairy and egg processing is their recovery for further use as human edible food. After food use, the value pyramid indicates that by-products should continue to be used in the food value chain, through their use in animal feed, followed by further industrial applications, while the production of bioenergy should come after upcycling in industrial applications. When all these options have been exhausted, the remaining by-products can be disposed of through combustion for energy or as a last resort land fill.

### Byproducts of dairy processing

The global dairy sector processed 930 million tons of milk in 2022 into a wide range of dairy products for human consumption and as fining agents for wine production (Cosme et al., 2012). The main by-product produced during milk processing is whey, from cheese as well as from Greek Yoghurt production (Pires et al., 2021), followed by skim milk, buttermilk, or ghee residue. Residuals and waste can include downgraded products, either due to defaults (e.g., damaged packaging, out of date, not meeting specification) or wastewater from cleaning manufacturing sites.

**Figure 2.**
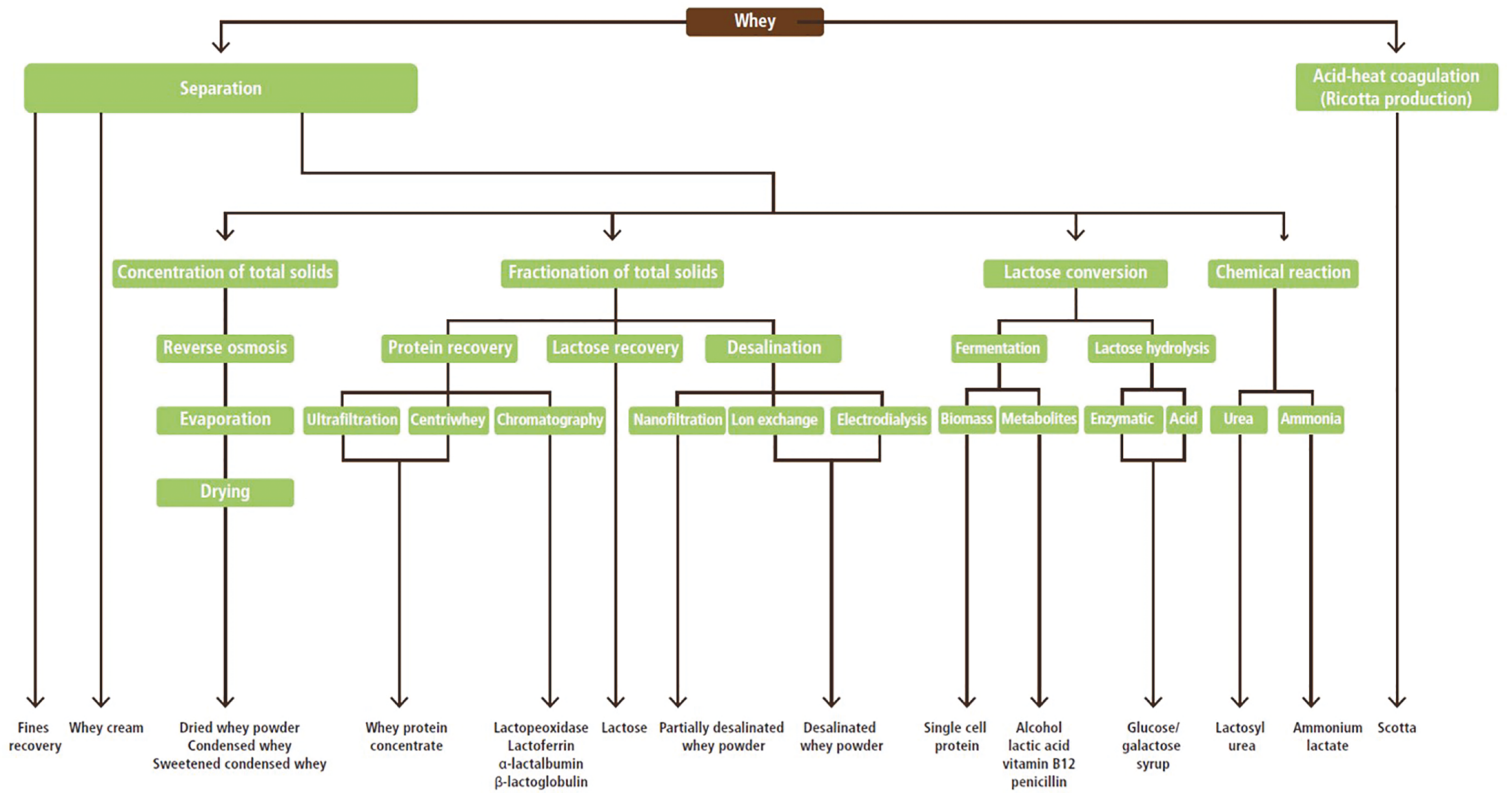
Flow diagram of potential co-products generated from processing whey (modified from Dairy Processing Handbook, 2023).

Whey is a dilute product containing lactose, soluble proteins, lipids, and minerals. On average, 9 liters of whey are produced out of 10 liters of processed milk (Buchanan, Martindale et al., 2023). The previous release of whey into the environment (i.e., discharge in waterways) was responsible for several significant pollution cases worldwide due to its high biological oxygen demand (BOD). Hence, there is now a legal mandate in most countries to prevent whey discharge, and many dairy processors apply technologies to concentrate the proteins, leading to the production of various co-products used in foods including infant formula, such as whey protein concentrate or isolate, lactalbumin and other co-products (Buchanan et al., 2023) (Figure 2) and water. Indeed, the protein content of whey is highly valued, and it is increasingly being recognized for its nutritional, medical, and human health benefits, where it is incorporated into whey protein beverages, including probiotic drinks. There has been a large and sustained increase in the use of these whey protein supplements, particularly focused on exercise, muscle-building, and sport outcomes, resulting in a current market worth ca US$8 billion (Keogh et al., 2019). These whey protein powder products have increased energy demand for production and are technically demanding, making them more suited for larger processors (Lavelli and Beccalli, 2022). Environmental impacts from the energy required for these products can be reduced when produced using high renewable energy sources. Despite the increased demand for whey protein powder, approximately 40% of global whey remains underutilized, although an increasing array of uses have been developed, including producing packaging and edible food coatings (Rafiq and Rafiq, 2019). Lactose, one of the main products from whey processing and protein extraction, is used in pharmaceuticals and in feeds (Rafiq and Rafiq, 2019).

**Figure 3.**
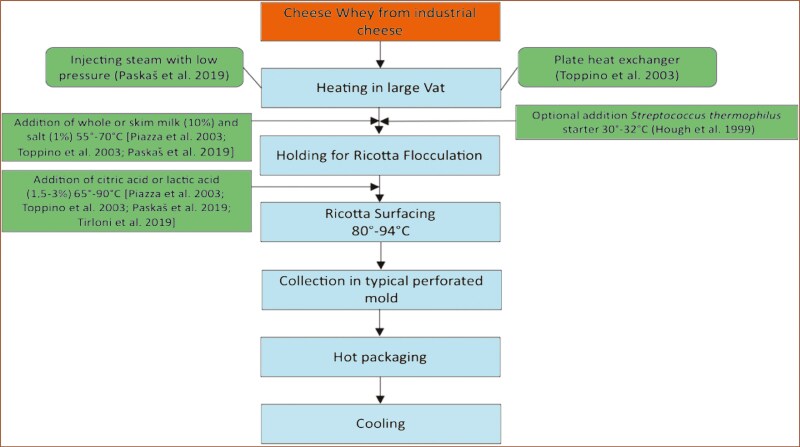
Production process of ricotta (Mangione et al., 2023). Brown: starting material, light blue: process description, green: other materials.

Sweet whey, resulting from the clotting of milk from various species, is directly used to produce cheese like ricotta, which is an unripened soft cheese produced originally in Italy (Mangione et al., 2023), using a specific production process (Figure 3). The ricotta yield from whey is highly variable (3.5 to 23% w/w) depending on the origin and composition of the sweet whey used (Mucchetti and Neviani, 2006). Ortiz-Araque et al. (2018) report yields ranging from 7.8 to 12% according to fat content, while other authors have reported yields of 5.3% (Streiff et al., 1979) and 6.0% (Salvatore et al., 2014), when non-condensed whey is used. Ricotta may be supplemented with cream or milk and may be considered as a fresh dairy product (Monti et al., 2018). About 850 kilotons of whey are used in Italy for ricotta, leading to the production of ricotta cheese exhausted whey (RCWE), a liquid by-product which can be used as a feed ingredient amongst other uses (Meo Zilio and Vasmara, 2025).

**Figure 4.**
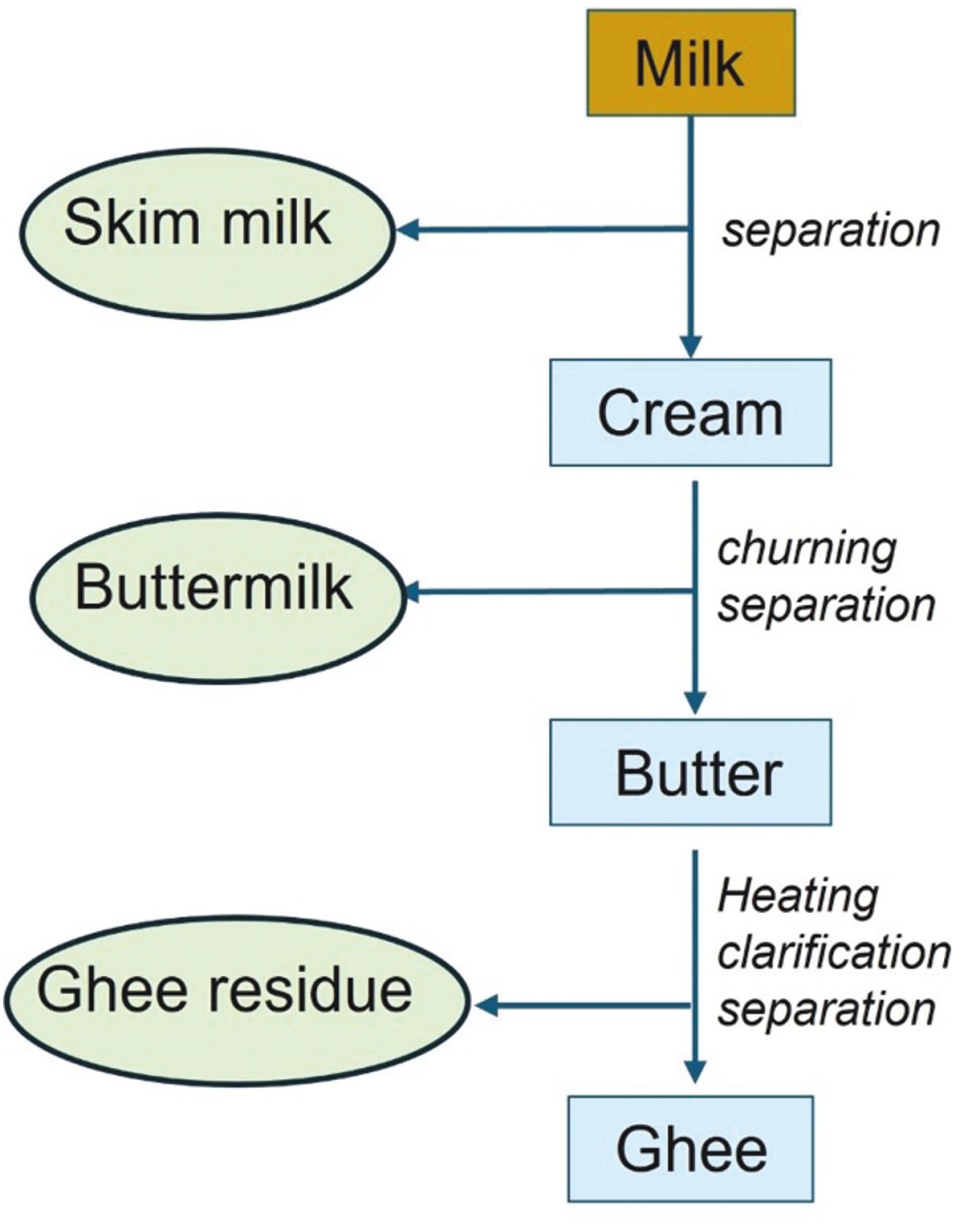
Simplified diagram of sources of production of buttermilk and ghee residue by-products. Note that there is a range of different ghee production methods (see Wani et al., 2022 for details). Orange: starting material, blue: products for human consumption, light green: byproducts.

Buttermilk and ghee residues are two other main by-products of the dairy industry (Figure 4), which are used for food applications in many parts of the world. Buttermilk is a by-product from butter production (more than 6 million tons generated from over 5 million tons of butter globally) and is used as a beverage, in baking, and in savory cooking. Its high phospholipid content makes it suited as an emulsifier in food systems, and it is used in a wide range of food products (e.g., confectionery, soups, bread, ice cream, and yoghurt), including in cheese spreads to increase functional properties (Rafiq and Rafiq, 2019).

Ghee is a clarified butter fat that is dominant in India (the world’s largest milk producer). About one-third of Indian total milk production is used to generate ghee by heating and desiccating butter. Ghee residue equates to about 10% of the ghee produced. This by-product has limited use in food industries for baking, flavoring, and confectioneries, while other potential applications include use for cosmetics, lipase production, animal feed ingredients, and biodiesel production, but most still currently go to waste in India (Wani et al., 2022).

Despite the various by-products from milk processing which are retained in the food industry, the steady increase in the vast production globally of cheese (over 22 million tons, currently), butter and ghee (over 10 million tons, currently) mean that there are still currently more by-products produced than are utilized in the food sector. Thus, it is essential for the sustainability of dairy industries globally that these by-products are further used down the value pyramid (i.e., for animal feed).

### Use of dairy byproducts in livestock feed

Dairy by-products represent an important potential source of livestock feed, but their nutritional value can vary widely, depending on the milk source and processing. Whey contains approximately 93% water, whereas the solids are composed of 70 to 72% lactose, 8-10% proteins, and 12 to 15% minerals (Rafiq and Rafiq, 2019). The bioavailability and quality of its constituents are high. Based on the digestible indispensable amino acids score (DIAAS), the protein quality is 1.1 to 1.6 for whey protein, that is, higher than most, if not all the plant-based protein feeds (0.4 to 0.9) (Rutherfurd and Moughan, 2012). Furthermore, the digestibility of whey dry matter in ruminants reaches up to 87% in diets that contain up to 30% whey.

Compared to whey, RCEW, sometimes referred to as scotta, is higher in ash, and the protein fraction is modified. This is related to the addition of acid and salt and the elevated temperatures used to produce ricotta. RCEW is rich in lactose and low in fat, with almost all the fat retained in the ricotta. The addition of salt may influence the mineral content of RCEW. While the concentration of protein is lower in RCWE compared to whey, it is highly digestible and contains a large amount of branched-chain essential amino acids (isoleucine, leucine, valine) and bioactive peptides (Chianese et al., 1997; Caroli et al., 2011). Furthermore, RCEW contains minerals (Ca, P, K, Mg, and Na), vitamin A, B-group, secondary metabolites, and minor compounds such as peptides, oligosaccharides, lactic acid, ketones, esters, free fatty acids, alcohol, and aldehydes (Fancello et al., 2024).

Buttermilk composition is generally comparable to skimmed milk with 32 to 33% protein, 49 to 54% lactose, and 6 to 13% fat on a dry matter basis (O’Callaghan, 2022). Ghee residues contain 12 to 27% moisture, 33 to 59% fat, 19 to 33% protein, 5 to 18% lactose, and 1.5% ash, with some variations depending on the source of the ghee residue (Wani et al., 2022).

Hence, dairy by-products are a valuable source of nutrients for livestock production (Modler et al., 1980) and have primarily been used in diets of pigs (Jedrejek et al., 2016) and poultry (Hilmi et al., 2020; Ferdous et al., 2025). However, due to its high-water content, it requires livestock production systems to be close to the dairy industry production sites.

### By-products of egg production

Laying hens are reared to produce eggs for human consumption. This is accompanied by two main by-products, manure and spent hens at the end of the production process, which may be used for meat production or rendered (Lee et al., 2025). In addition, around 10% of the eggs produced worldwide are rejected for human consumption and may be used as livestock feed (Al-Harthi et al., 2010).

Eggs are sold either intact or in liquid form. Approximately 30% of liquid eggs are used by the food industry (e.g., pasta, pastries), and the rest are used for manufacturing industrial products. After breaking, the egg is separated from the eggshell, the by-product of this process.

An eggshell is the outer part of the egg and is composed of the shell itself and membranes surrounding the albumen. This latter organic matter may be removed from the shell by calcination, treating the shell with sodium hydroxide (Faridi and Arabhosseini, 2018), or by separation, for further isolation of valuable components (Shellbrane Project, 2012; Ponkham et al., 2011). Assuming complete recovery of eggshell from global liquid egg production, Owuamanam and Cree (2020) estimated that more than 2 million tons of calcium carbonate could be produced for the replacement of mined calcium carbonate.

Eggshell membranes are composed of structural proteins (i.e., collagen and keratins) and other high-value proteins or peptides, such as lysozyme, ovotransferrin, ovalbumin, globulin, ovomucin, and defensin. These latter proteins play a role in the maintenance of a healthy digestive tract, thus potentially improving diet digestibility (Makkar et al., 2015).

## Use of egg by-products in livestock diets

Rejected eggs may be collected and dried, either with or without shell, after various processes (e.g., dried or boiled or autoclaved after freezing). Dried whole eggs are often ground to be incorporated into feed, e.g., broiler diets, to provide high-value amino acids, fatty acids, vitamins, and minerals (Al-Harthi et al., 2010; El-Deek et al., 2011).

Eggshell is composed of 94% calcium carbonate, and the eggshell membrane contains approximately 11% polysaccharides (of which one-third is predominantly chondroitin sulfate A and B); 3% fat and 70% protein (Leach Jr, 1982; Owuamanam and Cree, 2020). Hence, eggshells can be used as a source of calcium in livestock nutrition. Eggshell membranes have been evaluated in broiler chicken feeds at incorporation rates between 0.2 and 0.4% and were shown to improve growth performance. In addition, they were also shown to raise the concentration of immunoglobulin IgM and IgG in the plasma, indicative of an improved immune status, while reducing the concentration of blood cortisol, which suggested a stress reduction (Makkar et al., 2015). In heat-stressed quail, incorporation of 2% of dietary eggshell (including membrane) improved the oxidative status and maintained growth performance (Erisir et al., 2020).

## Other use of milk and egg by-products

When whey has not been processed for technological or economic reasons, it may be used as a fertilizer (Akay and Sert, 2020). However, many countries have strict regulations for this use. Alternatively, whey may be used directly or after protein extraction for anaerobic digestion (Casallas-Ojeda et al., 2021).

Wastewater (including from plant cleaning and spillage), downgraded products, scotta, and whey or whey extracts have also been used as substrates for microbial fermentation, biogas production, generating ethanol, lactic acid, solvents, surfactants, enzymes, biopolymers, bioplastics, hormones, vitamins, and bioactive compounds (Sar et al., 2022). Wastewater has also been subject to nanofiltration and reverse osmosis to recover water for on-site use, while the retentate has been added to fermented milk beverages and “dulce de leche,” formed from the heating of whole milk (Briao et al., 2019).

Eggshell may also be used in the production of biodiesel, acting as a catalyst for the transesterification of fatty acids (Faridi and Arabhosseini, 2018). Eggshell and its membrane may also be used for removal of heavy metals from water (Faridi and Arabhosseini, 2018), as construction material replacing calcium carbonate in cement (Veerabrahmam and Prasad, 2021), to produce hydroxyapatite for use in medical and dental applications (Faridi and Arabhosseini, 2018), or to produce various polymers (Owuamanam and Cree, 2020). Extracted eggshell membrane collagen has been used medicinally as joint supplements and has other biochemical, pharmaceutical, and cosmetic applications (Aina et al., 2022). Eventually, egg-derived products (egg white) are wine fining agents.

### Environmental benefits and risks

One kilogram of lactose, protein, and fat is equivalent to 1.1, 1.0, and 3.0 kg of chemical oxygen demand (COD), respectively (Ahmad et al., 2019). Therefore, whey has high COD (ca. 50 to 80 g/L) and BOD (ca. 40 to 60 g/L). Thus, it is a strong pollutant when released to waterways (Chatzipaschali and Stamatis, 2012). In the early 2000s, it was estimated that about 60% of whey was industrially processed to add-value products, while the rest was used for animal feeds, applied to land as fertilizer or disposed of in drains (Macwan et al., 2016). Consequently, many countries adopted legislation limiting whey disposal in waterways (Zandona et al., 2021). Hence, the increased use of whey in livestock production and other applications is necessary to further protect the environment, in a circular approach. Due to its rich organic load, scotta also has a high BOD and COD, which also represents a critical environmental risk, if not used as part of a circular bioeconomy.

**Figure 5.**
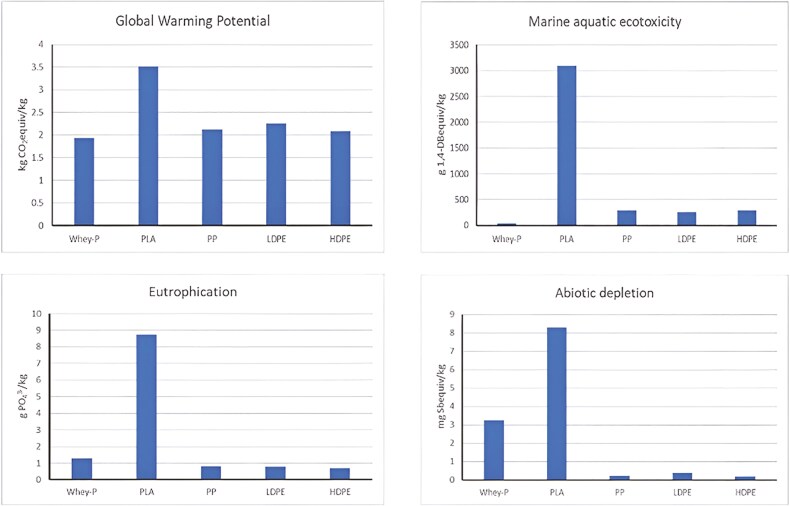
Comparison of the environmental impact of whey bioplastic versus fossil fuel plastics (Chalermthai et al., 2021). WheyP: whey bioplastic; PLA: plastic of polylactic; PP: fossil-based polypropylene; LDPE: low-density polyethylene; HDPE: high-density polyethylene.

Furthermore, Chalermthai et al. (2021) evaluated the environmental impact of the production of bioplastics by using whey protein compared to fossil-fuel-based plastics, using an attributional cradle-to-gate life cycle assessment approach. They demonstrated that the greenhouse gas emissions and marine ecotoxicity was reduced, when bioplastics were produced, while the eutrophication potential was comparable to other plastics and abiotic depletion was higher for whey bioplastics, due to energy use for copolymer production (Chalermthai et al., 2021) (Figure 5).

Industrial eggshell products are considered hazardous waste in some countries (Owuamanam and Cree, 2020). This is a further incentive for the collection of eggshells from industrial liquid egg processing plants for further use in the value chain. In Uganda and sub-Saharan Africa, localized collection schemes are utilizing eggshells for a range of uses, including as calcium supplements and building materials. The collection and further processing of eggshell thus reduces landfill waste and the associated risk for the environment. Furthermore, the use of eggshell as a source of calcium carbonate reduces the dependence on mined calcium carbonate, a finite resource, as a source of calcium for livestock production or for neutralizing acidic soils, reducing greenhouse gas emissions (Owuamanam and Cree, 2020).

## Conclusion

The processing of milk and eggs produces animal source food for human consumption and by-products, which can be further used within the value pyramid. Collecting whey, buttermilk and ghee residues from dairy production (approximately 200 million tons/year globally) and eggshell from egg processing operations (>2 million tons/year globally) enable the recovery of highly digestible and valuable nutrients (carbohydrate, protein, fats, and minerals) for human consumption, pharmaceutical, and industrial applications. A wide range of by-products have now been developed from whey for human consumption, but the large amount of whey produced means that its use for livestock production remains important.

The further use of dairy and egg by-products is a clear application of the circular bioeconomy principle and helps reduce the environmental footprint of livestock production systems.

## About the Authors

**Stewart Ledgard** is a Principal Scientist with AgResearch (a Crown Research Institute) and an adjunct Professor with the Life Cycle Management Centre at Massey University in New Zealand. His PhD covered nitrogen cycling in agricultural systems, and this research area expanded to cover impacts on water quality, resource use efficiency, and environmental emissions through agricultural supply chains. Stewart’s expertise covers detailed research in ruminant livestock farm systems and supply chains, with a focus on reducing emissions and increasing circularity, utilizing life cycle assessment. He has published over 280 research papers and 10 book chapters, as well as working closely with New Zealand farming, policy, and industry groups on reducing agricultural impacts on the environment and from livestock supply chains to international markets. Stewart has strong international collaborations and has regularly contributed to various guidelines and tools developed by the International Dairy Federation and the Livestock Environmental Assessment and Performance (LEAP) partnership.

**Michael Lee** FRSA FRSB FRASE ARAgS is an expert in sustainable livestock systems, defining their role in securing global food security at the same time as protecting environmental health (Livestock’s role in human and planetary health). He graduated with first-class honors in Animal Science from the University of Wales, Aberystwyth in 1997 and gained a PhD in ruminant nutrition (protein and energy metabolism) from the University of Aberdeen in 2001, followed by a Postgraduate certificate for teaching in higher education from Aberystwyth University in 2012. In November 2020, Michael moved from Rothamsted Research and the University of Bristol (where he held a joint position) to his current position as Deputy Vice Chancellor of Harper Adams University, England’s Premier specialist agriculture and land use University. He has published over 375 research articles and papers, including articles in Nature and Science. He served as President of the British Society of Animal Science from 2018 to 2022 and was a Trustee of the society for 12 years. In August 2019, he was elected President of the European Federation of Animal Science Livestock Farming Systems Commission. He represents the UK on the Animal Task Force, a Public-Private think-tank informing the EU parliament in Brussels, and is co-chair of the UK Universities Climate Network—Net Zero group. In 2023, he was selected to join a Technical Advisory Group of the UN’s Food and Agriculture Organization (FAO). Prof Lee also sits on the BBC’s Rural Affairs Committee, the Agriculture Advisory Group convened by the UK’s Climate Change Committee (CCC), and the Scientific Council of the World Farmers Organization (WFO).

**David Meo Zilio** is a Research Scientist in the role of the Italian Council for Agricultural Research and Economics (CREA), Research Centre for Animal Production and Aquaculture. He holds a PhD in Animal Production and Products’ Quality (University of Florence, Italy) with a background in meat science, animal welfare, and animal feeding and nutrition. Currently, his major interests are sustainable livestock production, methane emission measurements and reduction, and precision livestock farming. He is the Italian focal point for the Livestock Research Group of the Global Research Alliance and of the Global Methane Pledge Sector Agriculture.

**Philippe Becquet** is a consultant for regulatory and sustainability topics. He provides advice to the feed industry. Agronomist and animal nutritionist by background, Philippe Becquet has worked for more than 40 years in the feed industry at various positions and in various companies, accumulating experience and expertise in livestock production systems and the feed industry. He has also been active in feed industry associations such as IFIF (International Feed Industry Federation) and has contributed as an expert in the development of the Livestock Environmental Assessment and Performance (LEAP) guidelines on feed additives, the report on methane and more recently to the report on the role of livestock in circular bioeconomy.

**Corresponding author**: becquet@aphaia.fr
